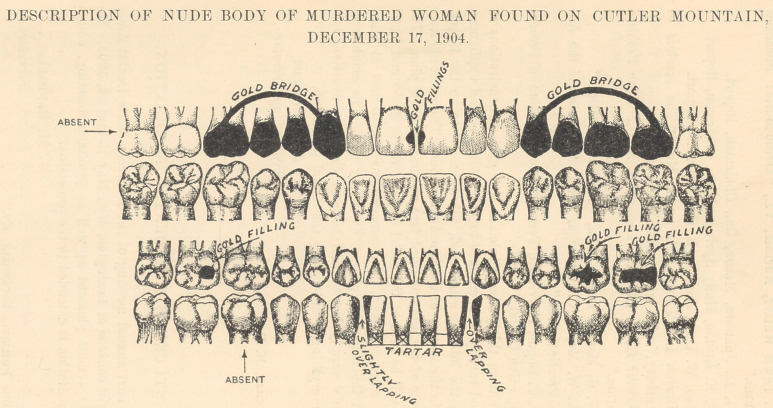# Current News

**Published:** 1905-02

**Authors:** 


### Current News.

### JOINT MEETING OF THE SOUTHERN BRANCH OF THE NATIONAL DENTAL ASSOCIATION AND TENNESSEE DENTAL ASSOCIATION.

THE eighth annual meeting of the Southern Branch of the National Dental Association will take place jointly with that of the Tennessee Dental Association at Memphis, Tenn., February 21 to 23, 1905. Special railroad rates, one and a third, certificate plan.

Following is a partial list of the papers and clinics:

Dr. Jules J. Sarrazin, New Orleans, La., Chairman, Report.

Dr. B. D. Brabson, Knoxville, Tenn., "Prophylaxis in Dentistry." Discussion opened by Dr. N. N. Vann, Atalla, Ala., followed by Dr. Robin Adair.

Dr. Robin Adair, Atlanta, Ga., "A Successful Introduction of Oral Prophylaxis Treatment into Practice." Discussion opened by Dr. N. N. Vann, Atalla, Ala., followed by Dr. B. D. Brabson, Knoxville, Tenn.

Dr. R. Boyd Bayle, Chairman, Report.

Dr. August F. Sonntag, Chairman, Report.

Dr. M. F. Fennily, Washington, D. C., Report.

Dr. E. P. Beadles, Danville, Va., "A Few Points in Inlay Work."

Dr. S. D. Ronebo, Marietta, Ga., "Gold and Tin and Amalgam and Gold at Cervical Margin as an Excellent Material for saving Teeth."

Dr. T. T. Moore, Columbia, S. C., "Insulating Deep-Seated Cavities."

Dr. B. Holly Smith, Baltimore, Md., title not given.

Dr. J. E. Chase, Ocala, Fla., Chairman, Report.

Dr. Geo. S. Vann, Gadsden, Ala., Chairman, Report.

Dr. F. M. Milam, Little Rock, Ark., "Orthodontia."

Dr. W. E. Grant, Louisville, Ky., "Orthodontia, Surgical and Mechanical."

Dr. J. Lewis Walker, Norfolk, Va., "Orthodontia: Successes and Failures."

Dr. H. H. Johnson, Macon, Ga., Chairman, Report.

Dr. R. K. Luckie, Holly Springs, Miss., Chairman, Report.

Dr. Geo. W. Dick, Sumter, S. C., Chairman, Report.

Dr. Burton Lee Thorpe, St. Louis, Mo., "The Masters of Early Dentistry," with lantern-slide pictures.

Dr. Arthur Hynes Fleming, Louisburg, N. C., "The Problem of Education."

Dr. W. G. Mason, Tampa, Fla., "Dental Education."

Dr. A. W. Meyer, Chattanooga, Tenn., "Diseases of the Antrum: A Practical Case."

Dr. J. C. Bogue, Harriman, Tenn., "The Education of Present and Prospective Dental Patients."

In addition thirty-nine clinicians will give demonstrations in an unusual variety of operations together with original appliances.

From the interest manifested, this promises to be the largest meeting in the history of the two Associations. The railroads have given a rate of one and one-third fare on the certificate plan. The meeting will be held at the Hotel Gayoso, rooms $1.50 and $2.00 per day, European plan. Accommodations can be had at other hotels on the American plan at $2.00 per day. The exhibits of the various supply houses will be exceptionally attractive, embracing everything of interest to the dental profession.

J. A. GORMAN,
*Corresponding Secretary.*

ASHEVILLE, N. C.

---

### KENTUCKY STATE DENTAL ASSOCIATION.

THE next annual meeting of the Kentucky State Dental Association will convene at Lexington, Ky., May 15 and 16, 1905. We anticipate a most pleasant as well as profitable meeting, and a cordial invitation is extended to the profession.

W. M. RANDALL,
*Secretary.*

MASONIC BUILDING, LOUISVILLE, KY.

DESCRIPTION OF NUDE BODY OF MURDERED WOMAN FOUND ON CUTLER MOUNTAIN, DECEMBER 17, 1904.

THE body was that of a woman well developed and apparently well kept, but discolored from fire and exposure to the elements. The face, nose, lips, chin, left side of neck, both ears, shoulders, and breasts were burned so as not to be recognizable

She was probably between twenty-five and thirtyfive years of age, weight about one hundred and twenty to one hundred and thirty pounds, height five feet two or three inches, light auburn or ash-blond hair, part of which was burned off; skin evidently fair, with no birth-marks or scars showing; small bones, limbs well rounded, hips and thighs large, very small hands, nails clean, long, and well manicured; feet small, toes even and straight, nails manicured; probably wore a No. 2, 2½, or 3 shoe.

*Teeth.*—The teeth were large, white, and chalky. In the upper jaw on the right side the wisdom-tooth had never developed; the second molar was present with no fillings. A bridge extended from the first molar to the cuspid. This bridge was of solid gold and worn on the linguo-mesial portion of the crown. The first and second bicuspid being absent, their places were supplied with solid dummies. Two gold fillings of medium size in the mesial of the upper centrals or incisors. The upper teeth protrude slightly. In the left upper jaw a gold bridge extended from the first bicuspid to the second molar; a peculiarity of this bridge is in the fact that the second molar is made of a bicuspid dummy. The third molar or wisdom-tooth on this side is present. In the lower jaw on the right side the third molar or wisdom-tooth is present; the second molar has a gold filling in the mesio-occlusal surface. The first molar is absent, evidently for some years, as the space is almost closed. Slight overlapping of cuspid on lateral. Pyorrhœa of lower teeth,—centrals and laterals,—with considerable tartar, showing that they had not been cleaned recently. Left side: considerable overlapping of cuspid on lateral; all teeth present on left side of lower jaw. First molar, large gold filling on occlusal surface; second molar, large gold filling on occlusal, extending on to the distal surface; third molar or wisdom-tooth undeveloped, that is, partially covered with tissue.

All clothing, finger- and ear-rings, and all other means of identification had been removed from the body, and no trace of same have been found, and up to the present time we have been unable to identify her.

The above description and diagram is the only evidence we have for identification.

Kindly call the attention of dentists in your city to the above description and diagram. If possible, have your newspapers print it.

Address all information and inquiries to

W. S. Reynolds,
*Chief of Police.*

Colorado Springs, Col., December 28, 1904.

### THE FRATERNAL DENTAL SOCIETY OF ST. LOUIS, MO.

At the annual election of the Fraternal Dental Society of St. Louis, December 20, 1904, the following officers for the ensuing year were elected:

President, Burton Lee Thorpe; Vice-President, E. P. Dameron; Secretary, S. H. Voyles; Treasurer, W. E. Brown.

*Executive Committee.*—E. E. Haverstick, W. L. Whipple, T. G. Donnell.

S. H. Voyles,
*Secretary.*

---

### A FREE DENTAL SERVICE AT THE HOMES OF THE SICK POOR.

The Dental School of Harvard University has recently organized its free dental service at the homes of the sick poor so as to be of greater efficiency than heretofore. The Dental School is now prepared to send a dentist, who is a graduate of the School and registered by the State Board of Registration, to the homes of the sick poor or to hospitals for the purpose of relieving pain originating from the teeth. This service does not extend to the filling of teeth or making of plates for artificial teeth. Its object is simply the relief of pain; a more extended treatment of the teeth being postponed until the patient may have recovered, and can visit the Infirmary of the School. The service here described can be obtained by telephoning the Harvard Dental School, North Grove Street, Boston, or by letter.

Eugene H. Smith,
Boston, November 1, 1904. *Dean.*

---

### GOLDEN ANNIVERSARY AT NEW ORLEANS.

On February 21, 1905, a golden anniversary banquet will be tendered by the dentists of New Orleans to Dr. George J. Friedrichs, who graduated in dentistry February 21, 1855.

L. D. Archinard,
*For Committee of Arrangements.*

## Figures and Tables

**Figure f1:**